# LncRNA UCA1 regulates immune micro-environment in cisplatin-induced AKI by miRNA-4498/AKT3 pathway

**DOI:** 10.1371/journal.pone.0314654

**Published:** 2025-02-12

**Authors:** Peng Hongjun, Lydia Mukanhaire, Liu Zhen, Wang Ting, Li Hongye, Zhang Xiaotian, Liu Guangling, Ren Xianguo

**Affiliations:** 1 Department of Pediatrics, Nanjing Drum Tower Hospital, Affiliated hospital of Medical School, Nanjing University, Nanjing, Jiangsu, China; 2 Jiangsu Key Laboratory for Biodiversity and Biotechnology, College of Life Sciences, Nanjing Normal University, Nanjing, Jiangsu, China; 3 Department of Pediatrics, Sir Run Run Hospital, Nanjing Medical University, Nanjing, Jiangsu, China; Fujian Provincial Hospital, CHINA

## Abstract

An increasing number of studies highlight the significance of long non-coding RNAs (lncRNAs) in the biological process of acute kidney injury (AKI). This study investigates the role and the mechanism of lncRNA UCA1 in cisplatin-induced AKI. Real-time quantitative PCR was used to measure lncRNA UCA1 expression in cisplatin-induced AKI mouse model, showing that lncRNA UCA1 was overexpressed. Knockdown of lncRNA UCA1 by shRNA significantly reduced inflammation caused by cisplatin treatment. A co-culture system demonstrated that lncRNA UCA1 upregulation in T cells induced apoptosis of tubular epithelial cells (TECs). A dual-luciferase reporter assay confirmed that lncRNA UCA1 acts as a miR-4498 sponge, binding to the 3’UTR of AKT3. Flow cytometry and ELISA results showed that reduced inflammation effect induced by lncRNA UCA1 knockdown was reversed by miR-4498 inhibition or AKT3 overexpression. Our findings suggest that lncRNA UCA1 functions as a miR-4498 sponge, upregulating AKT3 expression, and promoting inflammation in cisplatin-induced AKI.

## Introduction

Cisplatin-induced acute kidney injury (AKI) has become a growing health concern during cancer treatment in recent years [1]. The pathophysiology of cisplatin-induced AKI pathophysiology involves inflammation, apoptosis, tubular injury and vascular injury [2–5]. Recent research has increasingly focused on the role of T cells in cisplatin-induced AKI [6].

The family of long non-coding RNAs (lncRNAs) consists of transcripts longer than 200 nucleotides that do not code for proteins [7]. LncRNAs originate from conserved DNA regions [8] and may also be derived spliced exons of circular RNAs without open-reading frames (ORFs) [9–11]. It has been reported that lncRNAs are expressed in specific cell types, tissues and disease states. Their expression levels can vary across different stages of development. For example, IL-7R is highly expressed in *Homo Sapiens* [12], GAPLINC is specific in macrophages [13] and SENCR is selectively expressed in the endothelium [14]. In the immune system, lipopolysaccharide (LPS) induces the expression of lncRNA Mirt2, which can inhibit the NF-KB/ MAPK pathway through interaction with TRAF6 in macrophages [15].

LncRNA urothelial carcinoma-associated 1 (UCA1) is one of the cancer-related lncRNAs, initially discovered in 2006 as being overexpressed in bladder cancer cells [16]. It is located on chromosome 19p13.12, consisting of three exons and two introns, and belongs to the human endogenous retrovirus H family [16]. In adult human tissues, lncRNA UCA1 expression is relatively low, except in the spleen, placenta and heart [17]. LncRNA UCA1 is associated with the development gastrointestinal cancers and chemoresistance, as well as with hepatobiliary, pancreatic, and colon cancers [18]. However, the role of lncRNA UCA1 in cisplatin-induced AKI remains largely unexplored. Additionally, plasma exosomes from coronary artery disease patients show lower miR-4498 levels compared to healthy controls [19]. MiR-4498 regulates the inflammatory response by inhibiting TIMP3 expression in osteoarthritis [20]. This study focuses on elucidating the role of lncRNA UCA1/miR-4498/AKT in the immunological processes involved in cisplatin-induced AKI.

## Materials and methods

### Statement of ethics

This study’s protocol was reviewed and approved by Institutional Animal Care and Use Committee guidelines of Nanjing Medical University, approval number IACUC-036594.

### Animals

Experimental procedures were conducted on 10 to 14 weeks old male C57BL/6J wild-type (WT) mice, obtained from the Animal Core Facility of Nanjing Medical University. The animals were housed under a 12-hour light/dark cycle in air-conditioned rooms maintained at 22 ± 2°C and a relative humidity of 60 ± 10%. Before starting the experiments, the mice were given at least seven days to acclimate to these conditions.

### AKI model and experimental design

All mice were randomly divided into four groups (eight per group) as follows: sham + NC (normal saline), sham + shR, cisplatin + NC, and cisplatin + shR. To induce an acute kidney injury (AKI) model, the experimental mice were treated with a single dose of cisplatin (Sigma-Aldrich) administered by intraperitoneal injection (30 mg/kg), while control mice underwent a sham operation. The mice were anesthetized with pentobarbital sodium via intraperitoneal injection (45 mg/kg), and analgesia was provided by subcutaneous injection of buprenorphine (0.05 mg/kg). All mice were euthanized by cervical dislocation.

### Cell cultures and treatment

Cells were isolated from spleen tissues of cisplatin-induced AKI mice. CD4+ and CD25+ T cells were separated using a magnetic T cell Isolation Kit (Miltenyi Biotec, Germany). Cells were cultured in 1640 medium (Gibico, USA). Once T lymphocytes reached a suitable density, the cells were transfected with shRNA- UCA1, miR-4498 mimic, miR-4498 inhibitor (shR- lncRNA UCA1) (GenePharma, China), AKT3overexpression (AKT3 OE) and pcDNA-lncRNA UCA1(pcDNA-lnc) (Genewiz, China).

### Enzyme-Linked Immunosorbent Assay (ELISA)

Blood samples were collected from the mice and centrifuged to separate plasma. According to the manufacturer’s instructions, levels of the inflammatory factors’ interleukin 1 beta (IL-1β), tumor necrosis factor alpha (TNF-α), and interleukin 6 (IL-6) were measured.

### Fluorescence In Situ Hybridization (FISH)

FISH was used to determine the subcellular localization of lncRNA UCA1 (lncRNA FISH Probe Mix, GenePharma, China). T lymphocytes were cultured on a confocal plate. After reaching a certain intensity, cells were fixed with 4% paraformaldehyde. Cells were incubated overnight with a hybridization probe solution at 42°C. The nuclei were stained using DAPI, and the results were imaged using a fluorescence microscope (Olympus, Japan).

### Dual luciferase assay

The interactions among lncRNA UCA1, miR-4498 and AKT3 were confirmed. Wild type (WT) and mutant (MUT)versions of lncRNA UCA1 or AKT3were constructed and transfected (Genewiz, China). After transfection, luciferase activity was measured using a dual-luciferase reporter assay system (Promega, USA).

### Flow cytometry

Blood-derived leukocytes were divided into 4 groups: a T lymphocyte homotypic control group, and three experimental groups. The samples were incubated in the dark for 15 minutes then mixed with sheath solution to sort CD4+, and CD8+ cells. The proportions of CD4+/CD8+cells, and CD4−/CD8− cells were calculated.

### Western blot analysis

Cells were lysed with RIPA buffer (Beyotime, China), and western blotting was performed following standard protocols. The bands were incubated with AKT3 and GAPDH antibodies, and images were captured using Image Lab (Millipore, USA).

### TUNEL staining assay

Treated T cells were plated on an insert membrane, and tubular epithelial cells (TECs) were cultured on the well surface to create a co-culture system. TECs were fixed using 4% formaldehyde, permeabilized and, incubated with a TUNEL Apoptosis Assay Kit (Beyotime, China). The cell nuclei were then observed under a microscope.

### Statistical analysis

Statistical analyses were performed using SPSS v20.0. Student’s t-test or one-way ANOVA was used to determine statistical significance between groups. A p-value≤0.05 was considered statistically significant.

## Results

### LncRNA UCA1 expression increases in cisplatin-induced AKI mice

**Fig 1:**
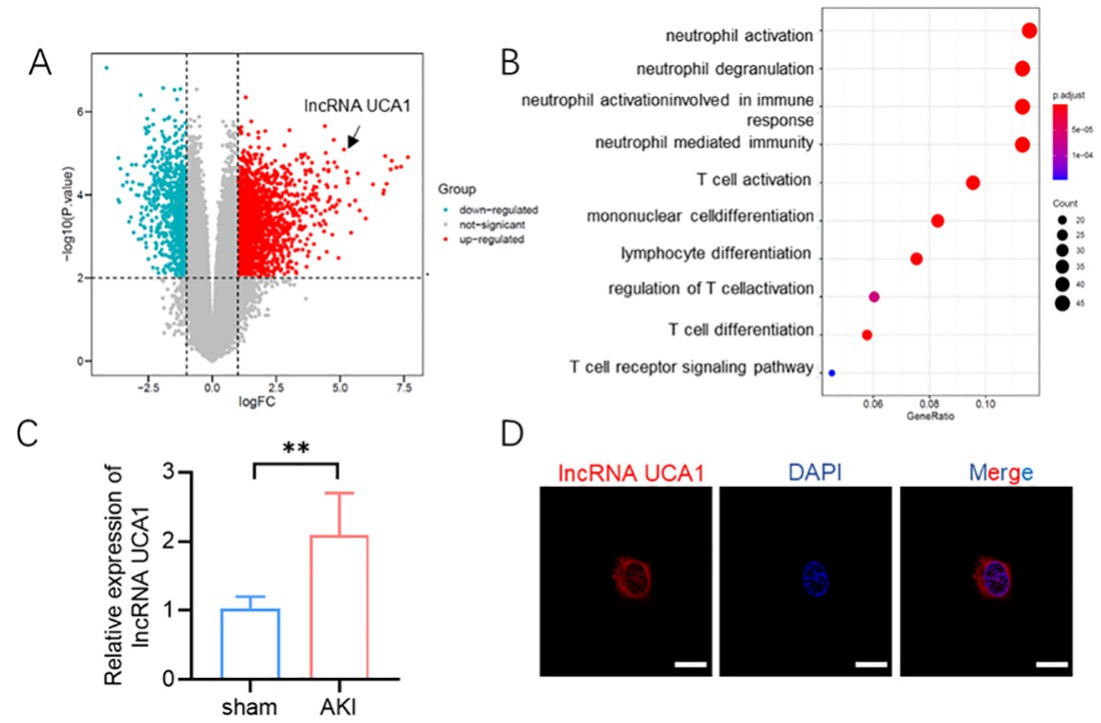
Identification of lncRNA UAC1 in cisplatin-induced AKI mice. **(A)** Volcano maps of differentially expressed lncRNAs in blood samples from cisplatin-induced AKI model obtained from GEO database (GSE142137). **(B)** KEGG pathways in differentially expressed lncRNAs. **(C)** RT-qPCR detection of lncRNA UAC1 levels in cisplatin-induced AKI mice serum. **(D)** Cellular localization of lncRNA UAC1 in T lymphocytes by FISH, Scar bar = 10μm. All values are represented by the mean ± standard deviation. *p<0.05, **p<0.01, ***P<0.001.

We utilized the GEO database to identify differently expressed lncRNAs in cisplatin-induced AKI models (Fig 1A). KEGG pathway analysis revealed that the differently expressed genes were enriched in the neutrophil activation pathway (Fig 1B). Using RT-qPCR. we detected elevated levels of lncRNA UCA1 in the blood of cisplatin-induced AKI mice compared to sham-treated mice (Fig 1C). FISH assay indicated that lncRNA UCA1 was primarily localized in the cytoplasm of T lymphocytes (Fig 1D).

### LncRNA UCA1 knockdown represses inflammation and T lymphocyte apoptosis in cisplatin-induced AKI mice

**Fig 2:**
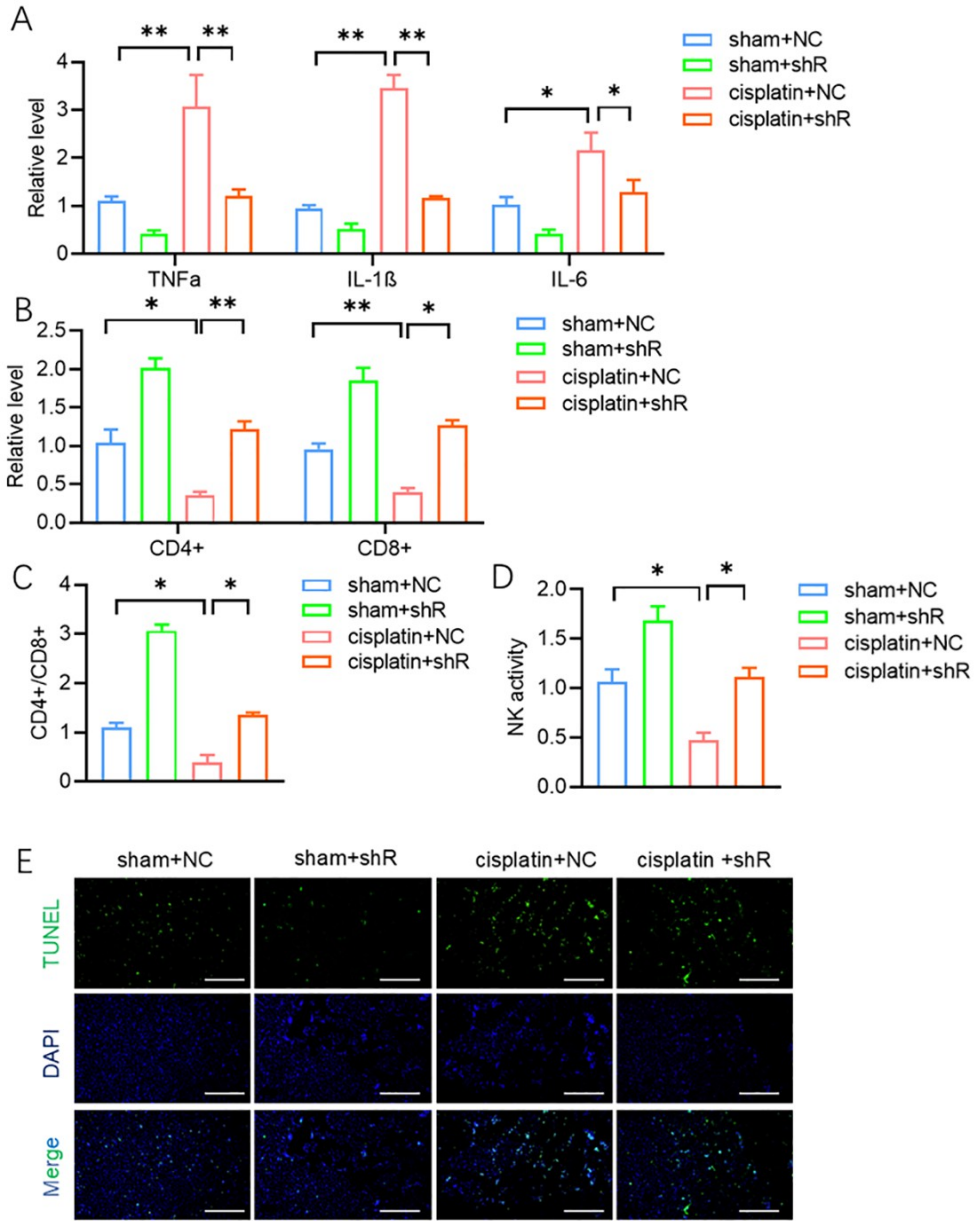
LncRNA UAC1 knockdown alleviates inflammation in cisplatin-induced AKI mice. **(A)** ELISA indicated the level of IL-1β, TNF-α, and IL-6 levels in serum in each group. **(B-D)** The proportions of T cell subsets and NK cells activity in each group was detected by flowcytometry. **(E)** TECs apoptosis examined by TUNEL staining in each group. All values are represented by the mean ± standard deviation. *p<0.05, **p<0.01, ***P<0.001.

To assess the function of lncRNA UCA1 in cisplatin-induced AKI we knocked down its expression by injecting shR- lncRNA UCA1 into the AKI mouse model. ELISA results showed that the levels of inflammatory cytokines IL-1β, TNF-α, and IL-6 were significantly reduced in mice treated with shR-1ncRNA UCA1 compared to the group (Fig 2A). Flow cytometry analysis indicated that of CD4+ and CD8+ cell proportions, as well as the CD4+/CD8+ ratio, decreased following lncRNA UCA1 knockdown (Fig 2B–2D). The TUNEL staining assay showed that lncRNA UCA1knockdown in T cells reduced tubular epithelial cells (TECs) apoptosis in the co-culture system (Fig 2E).

### LncRNA UCA1 targets miR-4498

**Fig 3:**
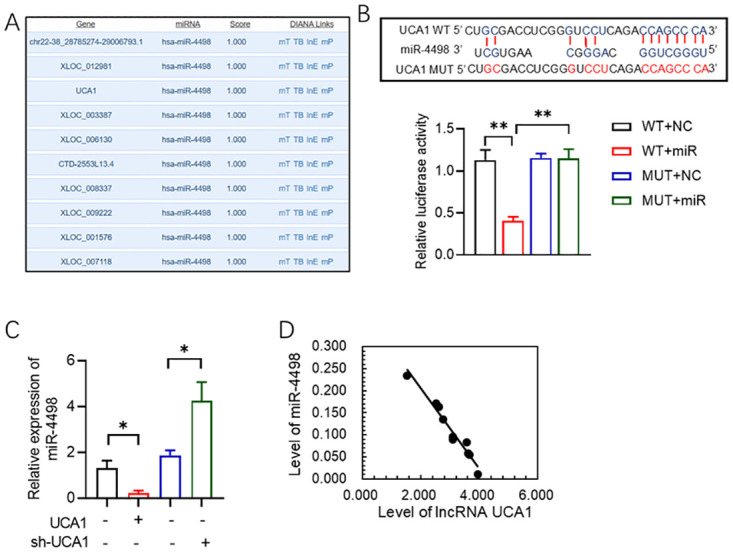
LncRNA UAC1 sponges miR-4498. **(A)** Micro-RNAs scores prediction by LncBase Predicted v.2. **(B)** Predicted miR-4498 binding sites in 3’UTR of lncRNA UCA1 and dual luciferase report assay confirmation. **(C)** Level of miR-4498 in T lymphocytes transfected with shR- lncRNA UCA1 or OE lncRNA UCA1. **(D)** Pearson’s correlation analysis between miR-4498 and lncRNA UCA1. All values are represented by the mean ± standard deviation. *p<0.05, **p<0.01, ***P<0.001.

To explore whether lncRNA UCA1acts as a miRNA sponge, we used LncBase Predicted v.2 to predict potential microRNA(miRNA) interactions. miR-4498 obtained the highest prediction score (Fig 3A). The dual-luciferase assay confirmed that miR-4498 binds to lncRNA UCA1 (Fig 3B). No significant luciferase activity changes were observed when the mutant (MUT) version of lncRNA UCA1 was transfected (Fig 3B). Further analysis revealed that miR-4498 levels were modulated by altering lncRNA UCA1 expression in T lymphocytes (Fig 3C). Pearson’s correlation analysis in the animal model demonstrated a negative correlation between miR-4498 levels and lncRNA UCA1 (Fig 3D).

### LncRNA UCA1 regulates inflammation through miR-4498/AKT3 axis

**Fig 4:**
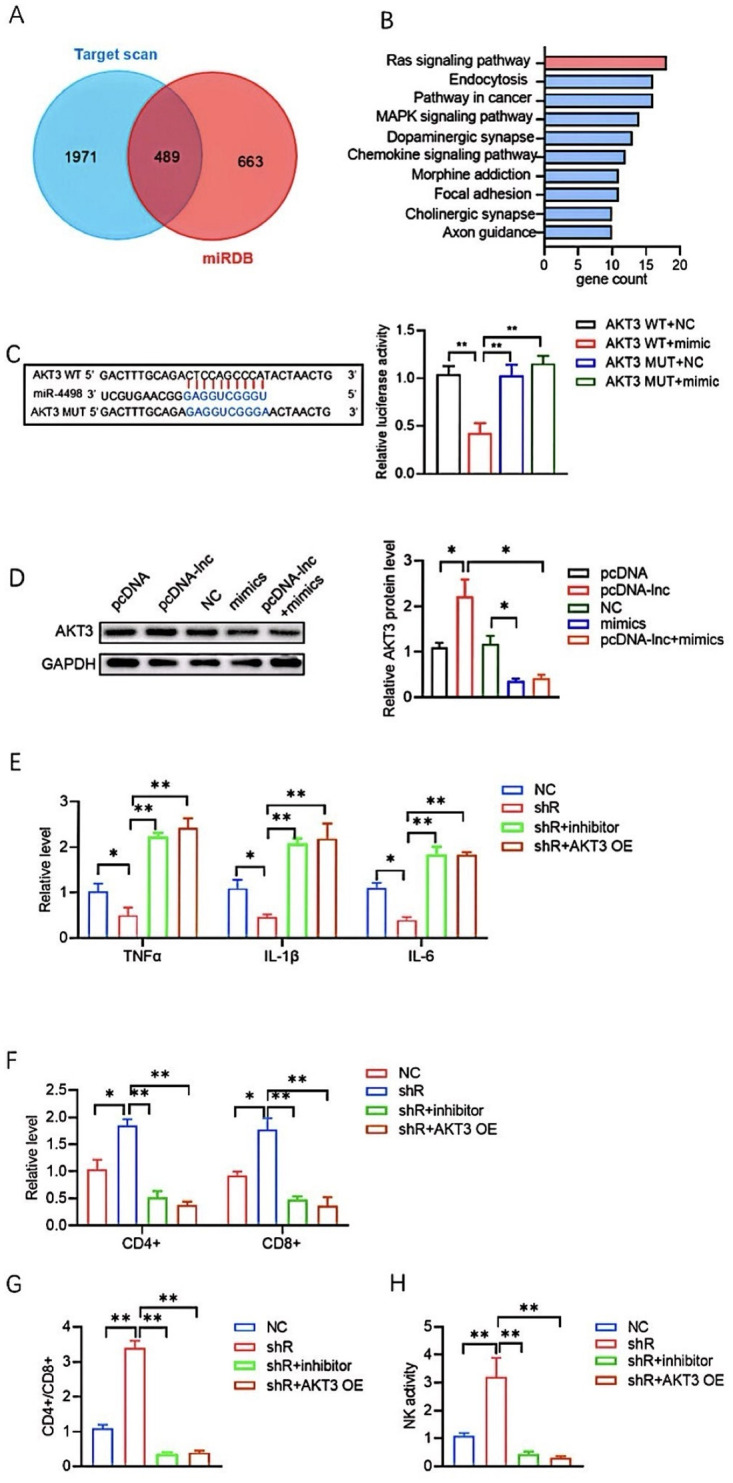
LncRNA UAC1 prompted inflammation through regulating miR-4498/AKT3 axis. **(A)** Two databases indicating the miR-4498 target genes. **(B)** KEGG pathway analysis for the same genes in two databases. **(C)** miR-4498 binding sites in AKT3 and dual luciferase assay. **(D)** AKT3 level detection by WB. **(E)** ELISA indicating levels of TNF-α, IL-1β, and IL-6in each group. **(F-H)** Proportions of T cell subsets and NK cells activity in each group as detected by flowcytometry. All values are represented by the mean ± standard deviation. *p<0.05, **p<0.01, ***P<0.001.

Using the bioinformatics databases, TargetScan and miRDB, we identified 489 common targets of miR-4498 (Fig 4A). KEGG pathway analysis revealed that these genes are enriched in the Ras signaling pathway (Fig 4B). Among the target genes, AKT3 was identifies as a key target of miR-4498. The dual-luciferase assay confirmed the binding affinity between miR-4498 and AKT39 (Fig 4C). Western blot analysis showed that the overexpression of lncRNA UCA1 significantly increased AKT3 expression, which was suppressed by miR-4498 mimics (Fig 4D). To test whether lncRNA UCA1 regulates inflammation through the miR-4498/AKT3 pathway, we transfected T lymphocytes with miR-4498 inhibitor or AKT3-OE along with shR- lncRNA UCA1. The anti-inflammatory effects observed in the shR- lncRNA UCA1 group were reduced after miR-4498 inhibition or AKT3-OE transfection (Fig 4E). Similarly, the reduced proportions of CD4+, CD8+ cells, and NK cells, as well as the lower CD4+/CD8+ ratio, in the shR-lncRNA UCA1 group, were restored after miR-4498 inhibition or AKT3-OE transfection (Fig 4F–4H).

## Discussion

In AKI, a substantial release of inflammatory cytokines from macrophages and other immune cells triggers a cascade of inflammatory responses, ultimately leading to multiple organ dysfunction [21]. Our study utilized a cisplatin-induced AKI model to investigate the role of the lncRNA UCA1/miR-4498/AKT3 pathway in mediating inflammation. We demonstrated that lncRNA UCA1 can act as a f miR-4498 sponge, and that its silencing reduced AKT3 expression, thus protecting against inflammation during cisplatin-induced AKI. In our study, the co-culture system revealed that lncRNA UCA1 overexpression in T cells induced apoptosis of tubular epithelial cells (TECs).

We used RT-qPCR to measure the expression levels of lncRNA UCA1 and miR-4498 in cisplatin-induced AKI mice. The results indicated that lncRNA UCA1 was highly expressed in serum of AKI mice, while miR-4498 levels were decreased. Previous studies have highlighted the importance of lncRNA UCA1 in various cancers, particularly gastrointestinal (GI) cancers and hepatocellular carcinoma (HCC) [22, 23]. It plays a crucial regulatory role in cell proliferation, migration and metastasis, especially in the context of chemoresistance [24]. Additionally lncRNA UCA1 is involved regulating immune responses, including the Th17 cell proportions and inflammatory cytokine production in acute ischemic stroke patients [25]. In this study, we verified that lncRNA UCA1 promotes inflammation in cisplatin-induced AKI by sponging miR-4498.

To explore the underlying mechanism, we employed bioinformatic databases and a dual luciferase assay, confirming that AKT3 is a direct target of miR-4498. The AKT family comprising of three related isoforms-AKT1, AKT2 and AKT3-plays critical roles in regulating cellular processes such as cell proliferation, immune responses [26–28]. Among the three isoforms, AKT3 is particularly important for inflammation and macrophage activation [26, 29]. Our results demonstrate that miR-4498 downregulates AKT3, while lncRNA UCA1 increases AKT3 expression by sponging miR-4498.

## Conclusion

We demonstrated that lncRNA UCA1 is significantly upregulated in cisplatin-induced AKI. Our findings show that the knockdown of lncRNA UCA1 effectively reduces inflammation and apoptosis in tubular epithelial cells through its regulation of the miR-4498/AKT3 axis. By acting as a sponge for miR-4498, lncRNA UCA1 increases AKT3 expression, which subsequently promotes inflammatory responses. These results suggest that lncRNA UCA1 plays a critical role in the pathogenesis of cisplatin-induced AKI and could serve as a novel therapeutic target for reducing inflammation and tissue damage in AKI. S1 and S2 Files provide additional context and validate the findings presented in this research.

## Supporting information

S1 File Spreadsheet containing experimental data for statistical analysis. Dataset collected during the experiment to measure in lncRNA UCA1 in cisplatin induced AKI mouse model and control mice.
(XLSX)

S2 File Raw experimental figures. Quantification of lncRNA UCA1 in cisplatin induced AKI mouse model and control mice.
(PDF)
