# Supplementary figures and images for: LncRNA UCA1 regulates immune micro-environment in cisplatin-induced AKI by miRNA-4498/AKT3 pathway
Source: PLoS One. 2025 Feb 12;20(2):e0314654. doi: 10.1371/journal.pone.0314654 (PMC11819503; doi:10.1371/journal.pone.0314654)

Figure 1D

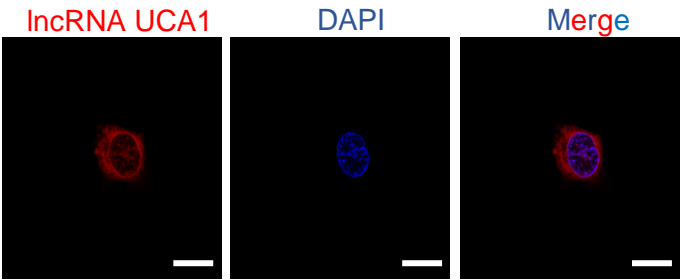

Figure2 E

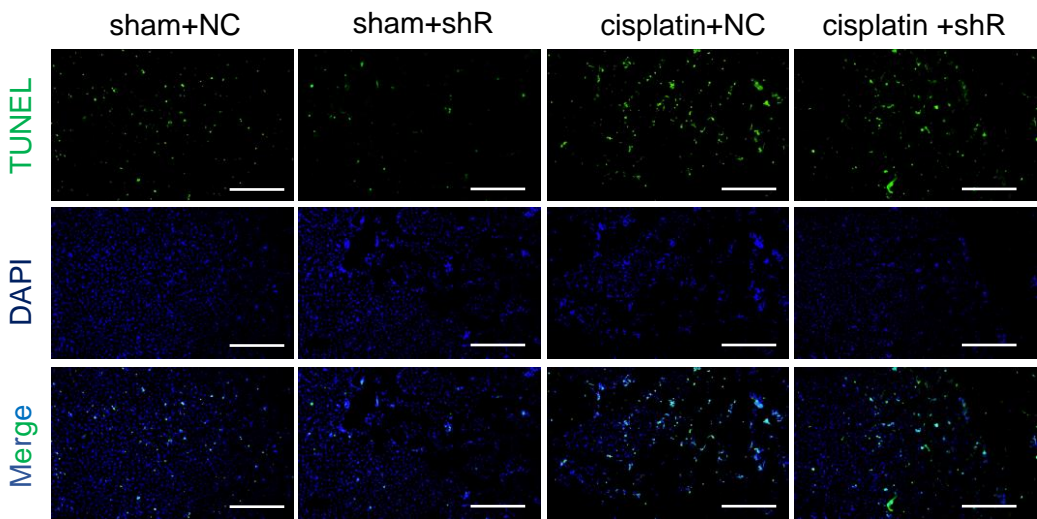

Figure 4D

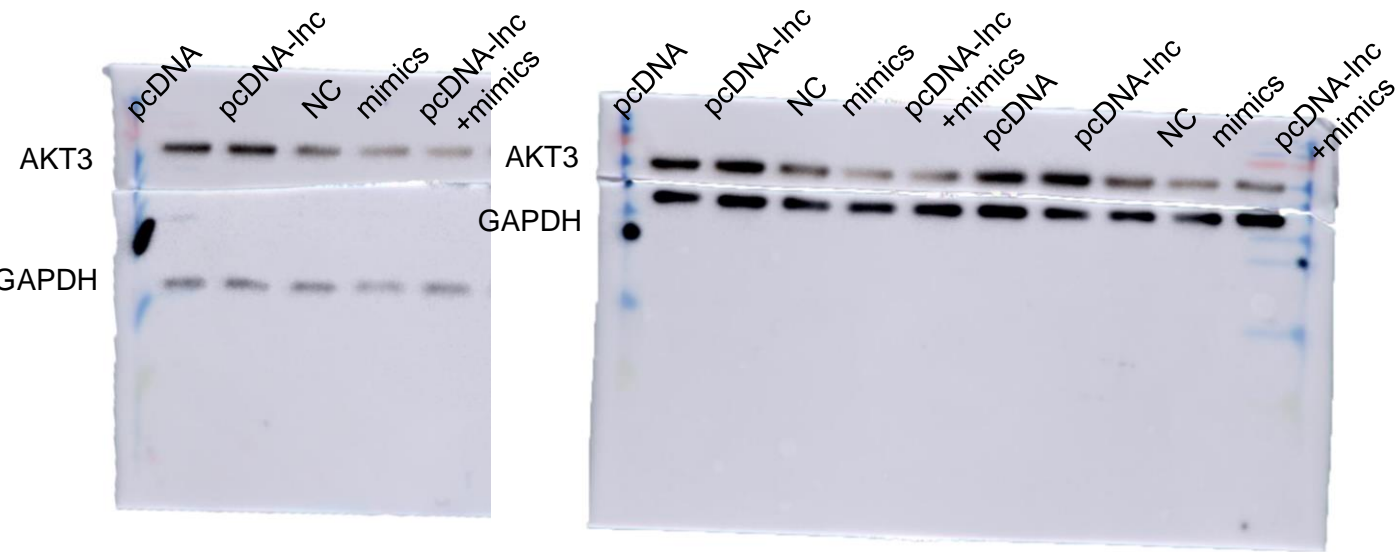

Supplement: S2 File — Quantification of lncRNA UCA1 in cisplatin induced AKI mouse model and control mice. (PDF) [file pone.0314654.s002.pdf]
